# Real-World Language Use With Familiar Versus Unfamiliar Interlocutors in Young and Older Adults

**DOI:** 10.1093/geroni/igaa057.2121

**Published:** 2020-12-16

**Authors:** Minxia Luo, Rudolf Debelak, Gerold Schneider, Mike Martin, Burcu Demiray

**Affiliations:** University of Zurich, Zurich, Zurich, Switzerland

## Abstract

Real-world contexts may compensate for age-related changes in language production. We compared age effects on vocabulary richness (i.e., entropy) and grammatical complexity (i.e., clause length) in conversations with familiar interlocutors (i.e., significant other, friends, family members) versus with strangers. We collected thousands of 30-seconds speech samples from 61 young and 48 healthy older adults across four days using a portable audio recording device — the Electronically Activated Recorder (EAR). Bayesian multilevel analyses showed that participants used richer vocabulary and more complex grammar with familiar interlocutors than strangers. Young adults used richer vocabulary than older adults. Furthermore, older adults produced equally complex grammar with the significant other as young adults did, but simpler grammar with friends and family members. We found no age group differences in grammatical complexity with strangers (lacking statistical power). In sum, familiarity with the significant other may benefit older adults in producing complex grammar in real-world conversations.

